# Is Serum Zinc Level Associated with Prediabetes and Diabetes?: A Cross-Sectional Study from Bangladesh

**DOI:** 10.1371/journal.pone.0061776

**Published:** 2013-04-17

**Authors:** Md. Rafiqul Islam, Iqbal Arslan, John Attia, Mark McEvoy, Patrick McElduff, Ariful Basher, Waliur Rahman, Roseanne Peel, Ayesha Akhter, Shahnaz Akter, Khanrin P. Vashum, Abul Hasnat Milton

**Affiliations:** 1 Centre for Clinical Epidemiology and Biostatistics (CCEB), School of Medicine and Public Health, The University of Newcastle, New Lambton Heights, New South Wales, Australia; 2 Department of Medicine, Mymensingh Medical College, Ministry of Health and Family Welfare, Government of Bangladesh, Mymensingh, Bangladesh; 3 Department of Biochemistry, Bangobondhu Sheikh Mujib Medical University (BSMMU), Dhaka, Bangladesh; 4 Department of Obstetrics and Gynaecology, Tairunnessa Memorial Medical College, Gazipur, Dhaka, Bangladesh; 5 Department of Paediatrics, Institute of Child and Mother Health (ICMH), Dhaka, Bangladesh; Brigham & Women's Hospital, and Harvard Medical School, United States of America

## Abstract

**Aims:**

To determine serum zinc level and other relevant biological markers in normal, prediabetic and diabetic individuals and their association with Homeostasis Model Assessment (HOMA) parameters.

**Methods:**

This cross-sectional study was conducted between March and December 2009. Any patient aged ≥30 years attending the medicine outpatient department of a medical university hospital in Dhaka, Bangladesh and who had a blood glucose level ordered by a physician was eligible to participate.

**Results:**

A total of 280 participants were analysed. On fasting blood sugar results, 51% were normal, 13% had prediabetes and 36% had diabetes. Mean serum zinc level was lowest in prediabetic compared to normal and diabetic participants (mean differences were approximately 65 ppb/L and 33 ppb/L, respectively). In multiple linear regression, serum zinc level was found to be significantly lower in prediabetes than in those with normoglycemia. Beta cell function was significantly lower in prediabetes than normal participants. Adjusted linear regression for HOMA parameters did not show a statistically significant association between serum zinc level, beta cell function (P = 0.07) and insulin resistance (P = 0.08). Low serum zinc accentuated the increase in insulin resistance seen with increasing BMI.

**Conclusion:**

Participants with prediabetes have lower zinc levels than controls and zinc is significantly associated with beta cell function and insulin resistance. Further longitudinal population based studies are warranted and controlled trials would be valuable for establishing whether zinc supplementation in prediabetes could be a useful strategy in preventing progression to Type 2 diabetes.

## Introduction

Diabetes Mellitus is a major public health problem for both developed and developing countries [1]. Considering the differences in risk factors across countries, it is estimated that the number of diabetics will be more than double by the year 2030 compared to 2000 [2], [3]. Most non insulin dependent diabetics and patients with impaired glucose tolerance are resistant to insulin stimulated glucose uptake [4].

Molecular and cellular studies in animal models have demonstrated that zinc (Zn) plays a key role in the synthesis and action of insulin under normal physiological conditions, and that zinc supplementation can be protective in rodent models of type 2 diabetes. In 1980, Coulston & Dandona demonstrated that zinc (II) chloride stimulated lipogenesis in rat adipocytes, similarly to insulin [5]. In another study of ob/ob mice, insulin secretion was potentiated following zinc supplementation [6]. Dietary zinc supplementation in young db/db mice for 6 weeks reduced fasting hyperglycemia and hyperinsulinemia [7].

In humans, there is a paucity of data about the role of zinc in insulin resistance. It is documented that diabetes patients have low serum zinc levels but this is likely to be due to hyperzincuria (losing zinc in the urine secondary to nephropathy in diabetes) and impaired zinc absorption [8]. Evidence for the causal role of zinc in the development of diabetes is circumstantial. In the islet cells of type 2 diabetes patients, Human Islet Amyloid Polypeptide (hIAPP) causes β cell loss and low insulin secretion; zinc significantly inhibits hIAPP amyloid fibrillogenesis and has been shown to prevent hIAPP mediated β cell loss [9]. Other studies in humans document the need for zinc in the synthesis and release of insulin from β cells [10], [11]. An observational study reported a lower incidence of type 2 diabetes in women who had higher intakes of dietary zinc [11]. Also, oxidative stress is relatively common in diabetes and zinc supplementation reduces oxidative stress [12]. Despite these data, a recent Cochrane review concluded that there was insufficient evidence to determine if zinc supplementation in adults is efficacious for prevention of insulin resistance [13]; another recent study suggested more detailed investigations of zinc supplementation on glucose metabolism [14].

Prediabetes often preceeds diabetes mellitus, and is characterized by impaired fasting glucose (IFG) and or impaired glucose tollerance (IGT) [15]. The condition is prevalent in many populations, especially among the middle aged and elderly [4], [16], [17]. Data from the United States estimates a prediabetic state among 40% of adults between 40 and 74 years [18], while an Australian study shows a 16.4% prevalence of prediabetes among adults 25 years of age or over [15]. The natural history of impaired fasting glucose (IFG) and impaired glucose tolerance (IGT) is variable; progression to diabetes occurs in approximately 25%, the abnormal glycemic state remains in 50%, and reversion to normal glucose tolerance (NGT) occurs in 25% over 3–5 years of observation [14]. Compared to normo-glycemic individuals, prediabetics are 5–15 times more likely to develop type 2 diabetes [18]. IFG and IGT also carry an increased risk of cardio vascular disease [19].

Given this existing data, it is reasonable to hypothesize that low zinc may predispose to insulin resistance and consequently, that zinc supplementation in prediabetes may prevent or delay progression to type 2 diabetes. Our study primarily aimed to evaluate whether serum zinc levels are associated with glycemic status, HbA_1_c, and HOMA parameters, i.e. β cell efficiency, insulin sensitivity and insulin resistancein normal, prediabetic and diabetic individuals

## Methods

This cross-sectional study was conducted in Bangobandu Sheikh Mujib Medical University (BSMMU), Dhaka, Bangladesh between March and December 2009. We obtained necessary ethics approval from the Ethics Committee, Director General Health Services, Ministry of Health and Family Welfare, Government of Bangladesh. Potential participants were approached by trained Study Medical Officers to participate in the study. After obtaining informed verbal consent the participants were recruited. With the low literacy rate noted in the planning phase of the study, we decided to obtain verbal consent from prospective participants and this was approved by the ethics committee. The Study Medical Officers maintained a register for those who consented verbally to participate in the study. In addition, at least 5% of the recruited participants were re-interviewed by the study investigators for quality control to cross check that verbal consent was properly obtained. Study participants were recruited from adults aged 30 or above attending the medicine outpatient department (MOPD) of the university hospital in weekly daytime business hours (9am to 2pm) for blood glucose testing and presented with or without any comorbidity other than signs and symptoms of glycemic conditions. After obtaining verbal consent and considering the participant's status (random or fasting), patients were screened by glucometer for their initial group allocation (Normal, Prediabetes and Diabetes) to aim for roughly equal numbers of participants in each group. The medical officers requested that participants come to the hospital on the following day for determination of their fasting blood glucose level in the laboratory. Subsequent analysis and group allocation in this paper are based on laboratory results of fasting blood glucose levels although we have taken out 25 participnats from normal and 25 participants from prediabetes group as they were on hypoglycemic agents at the time of fasting blood glucose measurement. The Study Medical Officers completed the data collection form on each participant on the day of screening by glucometer. A trained laboratory technician collected a blood sample (10 mls) from each study participant to analyse their fasting blood glucose, serum zinc, serum insulin, and serum HbA_1_C level.

### Exposure definitions and measurements

Considering the laboratory fasting blood glucose measurements, participants were categorized into three groups using American Diabetic Association (ADA) guidelines:

Normal (normoglycemic): fasting blood glucose level was <5.6 mmol/l,

Prediabetes: fasting blood glucose level was 5.6–6.9 mmol/l, and

Diabetes: fasting blood glucose level was ≥7 mmol/l.

#### Measurement of other exposure variables

Participants' self-reported socio-economic characteristics, smoking status, diabetes, hypertension, family history, medications (esspecially steroids), and anthropometric measurements were collected.

### Outcome measurement

#### Serum zinc measurement

Serum zinc level was measured using Atomic Absorption Spectro Photometry (AAS) (Shimadzu Corporation, Japan). Calibration curves for zinc were prepared using stock standard solution (1000 mg/l = 1000 ppm) with analytical grade zinc salt preserved in polypropylene bottles. There was a linear relationship between absorbance and concentration (r = 0.9993) throughout the calibration curve, and all results were obtained from the linear range. The samples were analyzed by graphite furnace Atomic Absorption Spectrometry (AAS) using the following settings: 213.9 nm of wavelength, 0.5 nm of slit width, BCG-D2 lamp mode, 8 mA of lamp with low current and 0 mA of lamp with high current.

### Statistical Analysis

Based on previous studies we assumed a mean difference of 2 mmol/L (±5 mmol/L) in serum zinc levels among three groups (normal, pre-diabetes and diabetes) and calculated a sample size of 300 with 95% confidence interval and 80% power. Assuming a 10% refusal and drop-out rate, recruiting a total of 330 eligible participants was estimated to be sufficient to observe the differences for this study, i.e. 110 in each group. Data were entered and analysed using STATA version 10.0 supplied by STATA Corporation, Texas, USA. Summary statistics were calculated and presented as mean, standard deviation and proportion by groups. Non parametric tests were performed for variables that did not follow a normal distribution. Scatter plots, simple and multiple linear regression analyses were performed to observe associations between serum zinc levels and glycemic status. Associations with zinc were undertaken with zinc treated as a continuous variable, and as a dichotomous variable (“normal” ≥700 ppb/L and “low” <700 ppb/L [20], [21]). Univariate regression analysis was performed to identify factors that were associated with participants' serum zinc status. Any factor that provided a univariate p value <0.25 was entered into a multiple regression model. Homeostasis Model Assesment (HOMA2) calculator (University of Oxford, UK website; http://www.dtu.ox.ac.uk/homacalculator/index.php) was used to calculate steady state beta cell function (%B), insulin sensitivity (%S) and insulin resistance (IR) for normal and prediabetes participants and multiple linear regression analysis was performed for all three HOMA parameters (beta cell function, insulin sensitivity and insulin resistance) to determine factors associated with each outcome.

## Results

Based on the laboratory fasting blood glucose levels, a total of 330 participants were initially recruited for this study; however, 25 participants from the normal group and 25 participants from the prediabetic group were dropped from subsequent analyses as they were already on hypoglycemic agents at the time of recruitment. Of the remaining 280 participants, 51%were normal, 13% had prediabetes and 36% had diabetes; charateristics of the participants are listed in Table 1. Participants were predominantly middle-aged, male, had at least high school education and were never smokers. Overall 22% of the participants had a positive family history of diabetes, 34% were hypertensive and 36% were obese. Approximately, 31% of the participants were on anti-hypertensive medications and 19% were taking any form of hypoglycemic agent. Mean fasting blood sugar levels were 4.3, 6.3 mmol/L and 11.9 mmol/L in normal, prediabetic and diabetic participants, respectively (Table 2).

**Table 1:** Characteristics of study participants by their glycemic status (N = 280).

Indicators	Normal (n = 143)	Prediabetic (n = 36)	Diabetic (n = 101)
**Age** (mean, ±SD):	45.5 (10.7)	46.7 (10.6)	47.4 (11.5)
**Sex** (n, %):			
Male	81 (56.6)	17 (47.2)	53 (52.5)
Female	62 (43.4)	19 (52.8)	48 (47.5)
**Education** (n, %):			
No education	18 (12.6)	8 (22.2)	10 (9.9)
Primary	17 (11.9)	4 (11.1)	11 (10.9)
Secondary/Higher secondary	67 (46.8)	15 (41.7)	52 (51.5)
Graduate/Masters	40 (28.0)	9 (25.0)	27 (26.7)
Others and missing	1 (0.7)	0 (0.0)	1 (1.0)
**Household income/m** (n, %):			
0–5000	36 (25.1)	3 (8.3)	10 (9.9)
5001–10000	39 (27.3)	16 (44.5)	32 (31.7)
10001–20000	43 (30.1)	10 (27.8)	35 (34.6)
>20000	24 (16.8)	7 (19.4)	22 (21.8)
No information	1 (0.7)	0 (0.0)	2 (2.0)
**Smoking status** (n, %):			
Smoked never	106 (74.1)	31 (86.1)	75 (74.3)
Previous smoker	19 (13.3)	1 (2.8)	18 (17.8)
Current smoker	18 (12.6)	3 (8.3)	8 (7.9)
No information	0 (0.0)	1 (2.8)	0 (0.0)
**Diabetes in family** (n, %):			
Yes	22 (15.4)	8 (22.2)	31 (30.7)
No	121 (84.6)	28 (77.8)	70 (69.3)
**Hypertension status** (n, %):			
Yes	43 (30.1)	17 (47.2)	32 (31.7)
No	80 (55.9)	13 (36.1)	52 (51.5)
Don't know	19 (13.3)	6 (16.7	16 (15.8)
No information	1 (0.7)	0 (0.0)	1 (1.0)
**BMI** (n,%):			
<18.5	15 (10.5)	1 (2.8)	8 (7.9)
18.5–25	77 (53.8)	24 (66.6)	55 (54.5)
>25	51 (35.7)	11 (30.6)	38 (37.6)
**Taking any medicines** (n, %):			
Yes	85 (59.4)	20 (55.6)	75 (74.3)
No	57 (39.9)	16 (44.4)	25 (24.7)
No information	1 (0.7)	0 (0.0)	1 (1.0)
**Taking antihypertensive** (n,%):			
Yes	43 (30.1)	17 (47.2)	28 (27.7)
No	85 (59.4)	11 (30.6)	65 (64.4)
No information	15 (10.5)	8 (22.2)	8 (7.9)
**Taking hypoglycaemics** (n, %)			
Yes	0 (0.0)	0 (0.0)	53 (52.5)
No	128 (89.5)	27 (75.0)	41 (40.6)
No information	15 (10.5)	9 (25.0)	7 (6.9)

**Table 2:** Laboratory findings of blood/serum analysis and Homeostasis Model Assessment (HOMA) using HOMA-2 calculator for beta cell efficiency of the participants in different groups (N = 280, Normoglycemic = 143, Prediabetic = 36 and Diabetic = 101).

Patient status	Laboratory findings				Beta cell efficiency using HOMA-2 calculator		
	Mean fasting blood glucose mmol/l ± SD (Median, IQR*)	Mean serum zinc,ppb/l ± SD (Median, IQR*)	Mean serum insulin,µu/ml ± SD (Median, IQR*)	Mean HbA_1_C level, % ± SD (Median, IQR*)	Mean % of betacell function ± SD (Median, IQR*)	Mean % of insulin sensitivity ± SD (Median, IQR*)	Mean insulin resistance ± SD (Median, IQR*)
**Normal (n = 143)**	4.3±0.60 (4.4, 4.0to 4.8)	585.31±160.63 (600, 500 to 700)	10.47±6.45 (9.3,6.2 to 14.0)	6.03±0.85 (6.0, 5.3 to 6.5)	154.69±78.24 (135.3, 99.6 to 185.2)	108.38±67.95 (88.1, 57.2 to 131.7)	1.30±0.76 (1.10, 0.8 to 1.7)
**Prediabetic (n = 36)**	6.3±0.38 (6.3, 6.0to 6.6)	520.55±161.36 (500,400 to 650)	11.33±9.15 (7.95, 6.65 to 12.15)	6.36±0.98 (6.3, 5.7to 6.85)	75.52±38.29 (63.65, 51.25 to 85.05)	90.27±43.95 (90.95, 60.8 to 107.95)	1.53±1.18 (1.10, 0.90 to 1.65)
**Diabetic (n = 101)**	11.87±5.3 (10.5, 8.3 to 13.7)	553.96±148.44 (550, 450 to 650)	15.89±19.0 (10.0, 6.8 to 17.6)	8.39±2.43 (8.0, 6.3to 9.7)	-	-	-
**Non-parametric Kruskal-Wallis P value**	φ<0.001, §<0.001, ψ<0.001	φ0.03, §0.02, ψ0.07	φ0.08, §0.8, ψ0.03	φ<0.001, §0.1, ψ<0.001	<0.001	0.4	0.4

* IQR = Interquartile range,

φ across all groups;

§ between normal and prediabetic only;

ψ between normal and diabetic only.

Zinc was significantly different between the disease groups, with the difference reaching statistical significance for the comparison between normal and pre-diabetes groups (P = 0.02, Table 2). This comparison remained statistically significant after adjusting for multiple possible confounders in a linear regression (Table 3), with the pre-diabetes group on average having a zinc level lower by about 66.7 ppb/l (P = 0.02) than normals. The results also remained consistent in multiple logistic regression when zinc levels were dichotomised at 700 ppb/l, a threshold used previously. We found that prediabetes and diabetes individuals were 78% (p = 0.008) and 54% (P = 0.01) less likely to have a normal serum zinc levels (≥700 ppb/L) compared to normal participants, respectively (data not shown). In all regression analysis, we included participants' gender and smoking status in the final model based on the findings from other studies [22], [23] although they were not associated in univariate analysis.

**Table 3:** Adjusted linear regression for serum zinc levels by participant's glycemic status ((N = 278, Normoglycemic = 142, Prediabetic = 35 and Diabetic = 101).

Exposure parameters	Serum Zinc		
	Coefficient	95% Confidence Interval	P Value
**Glycemic status:**			
Normoglycemic	Ref	Ref	Ref
Prediabetic	−66.69	−125.15 to −8.24	0.02
Diabetic	−25.60	−66.20 to 14.98	0.21
**Age**	−1.14	−2.96 to 0.67	0.21
**Sex**	−42.16	−86.37 to 2.04	0.06
**Education**	−2.44	−5.60 to 0.72	0.13
**Smoking History**	0.04	−31.26 to 31.34	0.99
**BMI**	2.24	−2.00 to 6.49	0.30
**Constant**	665.92	489.02 to 842.83	<0.001

In order to explore these associations more finely, we calculated the HOMA parameters. The HOMA is not suitable for use in frank diabetes and so these parameters were not calculated for the diabetes group.

We found that all these biochemical and HOMA parameters (Table 2) were non-normally distributed; therefore, the non-parametric Kruskal-Wallis test was used to test differences by glycaemic status. Mean serum zinc and mean serum insulin levels were significantly different between normoglycemic and prediabetes participants. Moreover, beta-cell function was significantly lower in prediabetic than in normal participants (P<0.001); however, there was no statistically significant difference in insulin sensitivity and insulin resistance levels between normoglycemic and prediabetic participants (P = 0.4) (Table 2).

All the HOMA parameters were then regressed against serum zinc levels as continuous values, stratified by the participant's glycemic status (Normal and prediabetes). We found that in normal people the higher the zinc the higher the insulin sensitivity and the lower the insulin resistance; as a result the less the beta cells have to function (hence the negative coefficient). These results are consistent whether zinc is analysed as a continuous or a dichotomised variable (Tables 4, 5, 6, 7). In prediabetic participants, similar stratified analysis did not show any significant association between HOMA parameters and serum zinc (Tables 5, 7)

**Table 4:** Adjusted linear regression analysis for HOMA parameters with serum zinc as continuous value in normal participants (N = 142).

Exposure indicators	HOMA2 Parameters								
	Beta Cell function			Insulin Sensitivity			Insulin Resistance		
	Coefficient	95% CI†	P value	Coefficient	95% CI†	P value	Coefficient	95% CI†	P value
**Serum Zinc**	−0.77	−0.15 to −0.00	0.05	0.040	−.02 to 0.10	0.19	−0.0008	−0.001 to −0.0001	0.02
**Age**	−0.90	−2.14 to 0.33	0.15	1.00	0.007 to 1.99	0.048	−0.006	−0.17 to 0.005	0.29
**Gender**	−6.54	−36.15 to 23.07	0.66	−15.38	−39.17 to 8.40	0.20	−0.067	−0.34 to 0.20	0.62
**Education**	−1.31	−4.12 to 1.50	0.36	−2.82	−5.07 to −0.56	0.015	0.002	−0.02 to 0.02	0.84
**Smoking History**	−1.36	−20.47 to 17.75	0.89	3.53	−11.82 to 18.88	0.65	−0.06	−0.23 to 0.11	0.49
**BMI**	7.17	4.29 to 10.05	<0.001	−6.13	−8.44 to −3.81	<0.001	0.08	0.05 to 0.11	<0.001
**Constant**	91.83	−40.97 to 224.64	0.17	229.13	122.45 to 335.82	<0.001	0.20	−1.02 to 1.44	0.74

† CI: Confidence Interval.

**Table 5:** Adjusted linear regression analysis for HOMA parameters with serum zinc as continuous value in prediabetic participants (N = 35).

Exposure indicators	HOMA2 Parameters								
	Beta Cell function			Insulin Sensitivity			Insulin Resistance		
	Coefficient	95% CI†	P value	Coefficient	95% CI†	P value	Coefficient	95% CI†	P value
**Serum Zinc**	−0.01	−0.09 to 0.06	0.78	0.02	−0.07 to 0.12	0.66	0.000	−0.002 to 0.002	0.89
**Age**	−0.45	−1.75 to 0.85	0.48	0.39	−1.24 to 2.03	0.62	−0.12	−0.05 to 0.02	0.55
**Gender**	0.83	−30.11 to 31.77	0.96	−4.12	−43.15 to 34.79	0.82	0.000	−0.97 to 0.97	1.00
**Education**	0.009	−2.55 to 2.56	0.99	−0.02	−3.24 to 3.20	0.98	−0.001	−0.08 to 0.07	0.97
**Smoking History**	−8.56	−32.28 to 15.15	0.46	13.24	−16.62 to 43.11	0.37	−0.28	−1.02 to 0.46	0.45
**BMI**	3.64	0.68 to 6.61	0.018	−2.57	−6.30 to 1.16	0.17	0.11	0.01 to 0.20	0.02
**Constant**	22.20	−108.91 to 153.31	0.73	114.45	−50.67 to 279.58	0.17	−0.33	−4.47 to 3.80	0.87

† CI: Confidence Interval.

**Table 6:** Adjusted linear regression analysis for HOMA parameters with serum zinc as category in normal participants (N = 142).

Exposure parameters	HOMA2 Parameters								
	Beta cell function			Insulin sensitivity			Insulin resistance		
	Coeff.*	95% CI*	P value	Coeff.*	95% CI*	P value	Coeff.*	95% CI*	P value
**Zinc categories (≥700 ppb/L)**	−28.51	−54.47 to −2.56	0.03	16.13	−4.72 to 36.99	0.12	−0.35	−0.59 to −0.11	0.004
**Age**	−0.88	−2.1 to 0.34	0.16	0.99	0.005 to 1.98	0.049	−0.006	−0.01 to 0.005	0.28
**Gender**	−4.50	−33.76 to 24.75	0.76	−16.36	−39.88 to 7.15	0.17	−0.05	−0.32 to 0.21	0.71
**Education**	−1.52	−4.35 to 1.29	0.28	−2.68	−4.95 to −0.41	0.02	−0.000	−0.02 to 0.02	0.97
**Smoking History**	−0.08	−19.18 to 19.01	0.99	2.80	−12.55 to 18.15	0.72	−0.04	−0.22 to 0.13	0.60
**BMI**	7.45	4.54 to 10.36	<0.001	−6.30	−8.64 to −3.96	<0.001	0.08	0.06 to 0.11	<0.001
**Constant**	46.70	−76.36 to 169.78	0.45	252.91	153.99 to 351.83	<0.001	−0.27	−1.40 to 0.86	0.63

* Coeff: Coefficient,

* CI: Confidence Interval.

**Table 7:** Adjusted linear regression analysis for HOMA parameters with serum zinc as category in prediabetic participants (N = 35).

Exposure parameters	HOMA2 Parameters								
	Beta cell function			Insulin sensitivity			Insulin resistance		
	Coeff.*	95% CI*	P value	Coeff.*	95% CI*	P value	Coeff.*	95% CI*	P value
**Zinc categories (≥700 ppb/L)**	−20.86	−59.33 to 17.6	0.27	16.84	−32.33 to 66.02	0.48	−0.55	−1.77 to 0.66	0.35
**Age**	−0.44	−1.72 to 0.83	0.48	0.39	−1.24 to 2.02	0.62	−0.01	−0.05 to 0.02	0.56
**Gender**	0.17	−29.94 to 30.29	0.99	−3.07	−41.57 to 35.43	0.87	0.002	−0.95 to 0.95	0.99
**Education**	−0.12	−2.62 to 2.37	0.92	0.12	−3.07 to 3.32	0.93	−0.003	−0.08 to 0.07	0.92
**Smoking History**	−7.70	−30.66 to 15.24	0.49	11.94	−17.39 to 41.39	0.41	−0.27	−1.00 to 0.44	0.43
**BMI**	3.62	0.72 to 6.52	0.01	−2.57	−6.28 to 1.13	0.16	0.10	0.016 to 0.20	0.02
**Constant**	20.20	−102.18 to 142.60		122.59	−33.86 to 279.06	0.12	−0.16	−4.03 to 3.71	0.93

* Coeff: Coefficient,

* CI: Confidence Interval.

**Figure 1:**
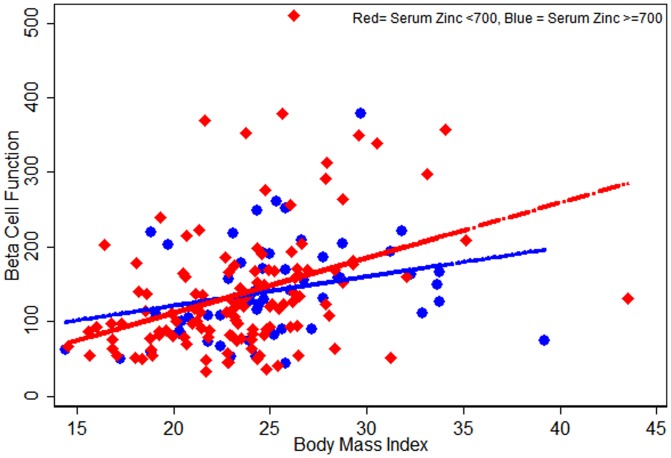
Scatter plots for HOMA-Beta Cell Function against Serum Zinc levels (<700, ≥700) by participants Body Mass Index (n = 179; normal = 143 and prediabetic = 36).

**Figure 2:**
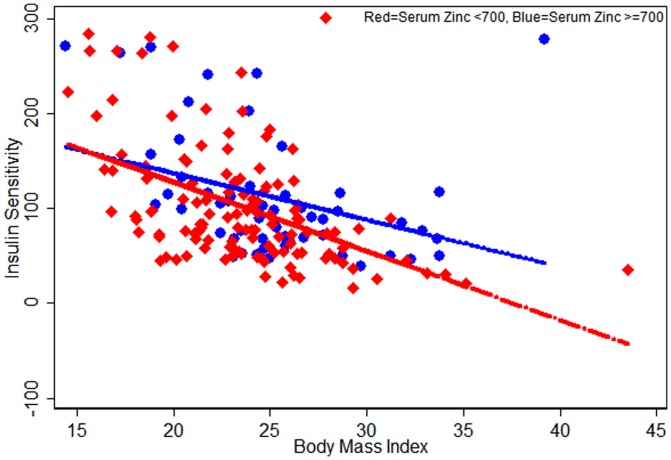
Scatter plots for HOMA-Insulin Sensitivity against Serum Zinc levels (<700, ≥700) by participants Body Mass Index (n = 179; normal = 143 and prediabetic = 36).

**Figure 3:**
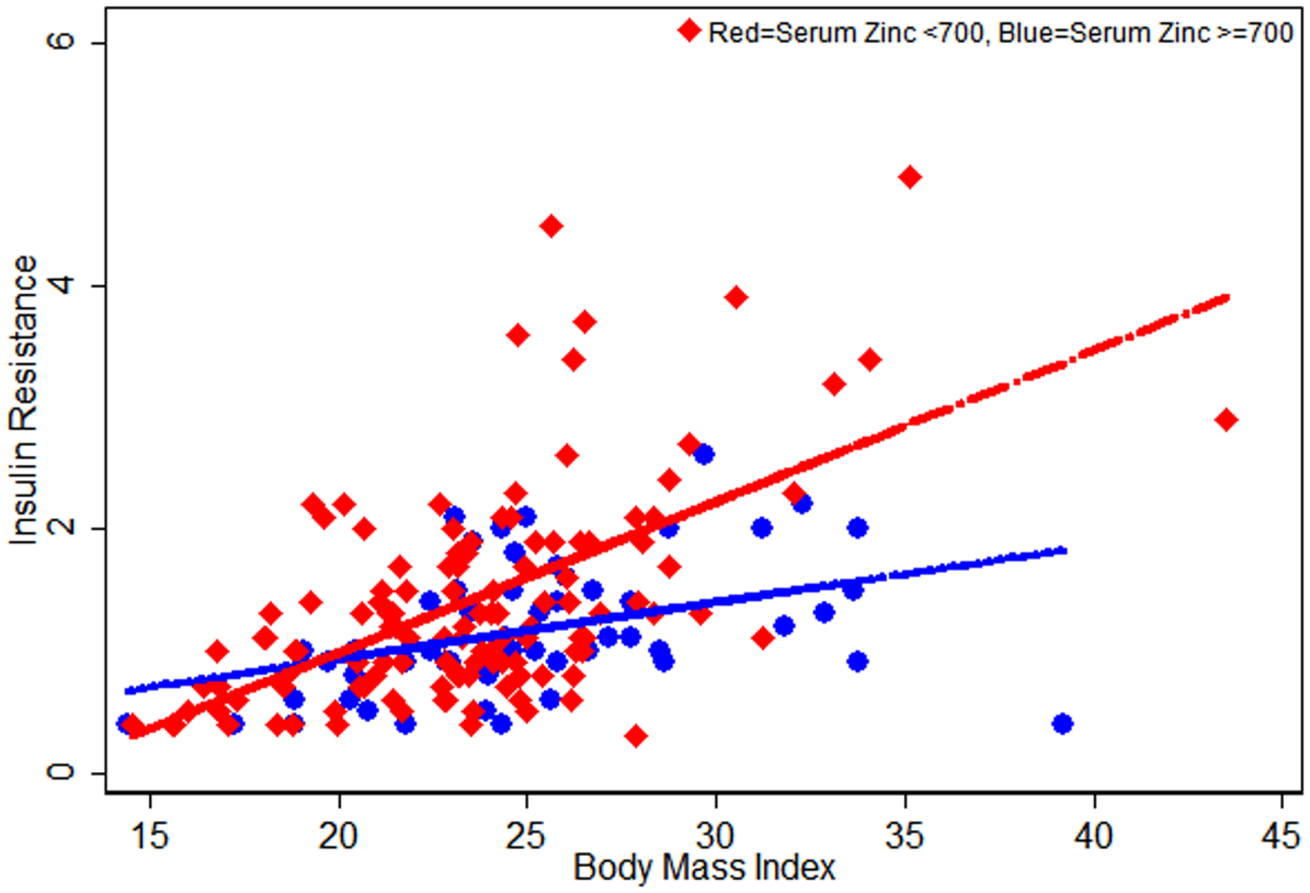
Scatter plots for HOMA-Insulin Resistant against Serum Zinc levels (<700, ≥700) by participants Body Mass Index (n = 179; normal = 143 and prediabetic = 36).

Given the strong effect of BMI on diabetes risk, we also explored the effect of BMI on HOMA parameters and zinc. Figures 1, 2, 3 indicates that serum zinc as a dichotomous variable has an interaction with BMI; we observed a significant interaction between dichotomized serum zinc and BMI for insulin resistance in multivariate analysis (P for interaction = 0.005) (Figure 3). At the same high BMI, participants with low serum zinc levels were more likely to have higher insulin resistance than those with high serum zinc levels (Figure 3).

## Discussion

Our study clearly indicates that mean serum zinc levels were the lowest in the prediabetes participants and this remained significant even when adjusted for multiple potential confounders. Even though this is a cross-sectional study, we recruited participants at different stages in the diabetic spectrum; the results suggest that low zinc precedes the development of diabetes and that low zinc is not simply an epiphenomenon of increased renal loss of zinc in diabetics. This has not been evident in previous cross-sectional studies. To further explore the role of low zinc in this process, we calculated the HOMA parameters. We found that higher zinc was associated with lower beta cell function and lower insulin resistance, and this was statistically significant regardless of whether zinc was treated as a continuous or a dichotomized variable. There was also an interaction between dichotomized zinc and BMI, indicating that at any given BMI, those with low zinc had higher insulin resistance than those with high zinc.

Other results were more puzzling. Stratified analyses in the pre-diabetic group did not show any effect of BMI or zinc, in contrast to the results in the normoglycaemic group. This may simply be due to insufficient power in our analysis or may be a genuine difference due to the fact that in prediabetes, participants may have reached to a metabolic break point.

Globally, zinc deficiency is widespread and 33% of the world's population are affected [24]. Studies report that diets rich in phytates and dietary fibers which contain low sources of readily bioavailable zinc can cause zinc deficiency in individuals and populations [25], [26]. In rural Bangladesh, predominant consumption of rice based diet with a few animal foods are the main risk factors for zinc deficiency [27]. Our finding of low zinc among pre-diabetes participants without significant renal disease argues that low zinc precedes the diabetes state and is not simply a result of renal loss of zinc, indicating that zinc may play a substantial role in the progression of this disease. There are many potential mechanisms for this; zinc is important in regard to metabolic diseases (insulin resistance, metabolic syndrome and diabetes) mainly because it is required for insulin storage in pancreas and stabilizing of insulin hexamers. Its anti-oxidative properties may delay progression of insulin resistance and diabetes [28]. Besides, reduction in some particular forms of zinc such as zinc-α2-glycoprotein (ZAG), an adipokine, plays a role in the development of metabolic diseases [29].

In previous studies, higher body mass index was found to be related with insulin resistance with or without other associated conditions [30], [31], [32]. Release of chemokines and inflammatory cytokines from adipose tissue in obesity may cause chronic systemic low grade inflammation, thus insulin resistance may develop [30]. In obesity, blood zinc levels were found to be lower and inversely related to obesity [22] and the level was significantly lower than controls [20]. One study demonstrates that, alteration in the concentration of zinc in obesity may contribute to the development of insulin resistance as zinc improves the solubility of insulin in the beta cells of pancreas and increases the capacity of the receptor for binding this hormone. They also recommend further research on the metabolic role of zinc in the insulin resistance syndrome [21] and short term metabolic disorders may not be associated with low plasma zinc in obesity [23].

Our study has a number of potential limitations:

- We do not consider other trace elements such as iron and copper that may influence participant's zinc status.
- In this study we used a glucometer to screen adults who attended for blood sugar analysis in the study hospital's medicine outpatient department. Our aim for screening was to obtain an equal number of participants in each group. The use of a glucometer for measuring blood sugar levels has previously been found to have a low sensitivity for correctly detecting an individual's blood sugar level [33], [34] and laboratory venous blood sugar measurement has since been recommended over the preference of bedside capillary blood sugar measurement using a glucometer [35]. Similarly, our study also observed differences in the results of blood sugar levels using glucometer and laboratory measurements and therefore, the number of participants recruited was different across groups.
- Our study is cross-sectional and we infer progression from the results of those in various groups, i.e. normal, pre-diabetic, and diabetic. These results need to be replicated in longitudinal studies.

In conclusion, prediabetic individuals are more likely to be zinc deficient and zinc deficiency in turn is associated with poorer beta-cell function and greater insulin resistance. There is an interaction with BMI in that for any given BMI, those with low zinc are more insulin resistant than those with high zinc. The results suggest the potential of targeting zinc supplementation for those with pre-diabetes or who have high BMI to prevent or impede the progression to diabetes. Further population based research and prospective controlled trials are required to clarify these findings.
